# Associations Between Night Work and Anxiety, Depression, Insomnia, Sleepiness and Fatigue in a Sample of Norwegian Nurses

**DOI:** 10.1371/journal.pone.0070228

**Published:** 2013-08-07

**Authors:** Nicolas M. F. Øyane, Ståle Pallesen, Bente Elisabeth Moen, Torbjörn Åkerstedt, Bjørn Bjorvatn

**Affiliations:** 1 Department of Public Health and Primary Health Care, University of Bergen, Bergen, Norway; 2 Norwegian Competence Center for Sleep Disorders, Haukeland University Hospital, Bergen, Norway; 3 Department of Psychosocial Science, University of Bergen, Bergen, Norway; 4 Department of Clinical Neuroscience, Karolinska Institutet, Stockholm, Sweden; San Francisco Coordinating Center, United States of America

## Abstract

**Background:**

Night work has been reported to be associated with various mental disorders and complaints. We investigated relationships between night work and anxiety, depression, insomnia, sleepiness and fatigue among Norwegian nurses.

**Methods:**

The study design was cross-sectional, based on validated self-assessment questionnaires. A total of 5400 nurses were invited to participate in a health survey through the Norwegian Nurses' Organization, whereof 2059 agreed to participate (response rate 38.1%). Nurses completed a questionnaire containing items on demographic variables (gender, age, years of experience as a nurse, marital status and children living at home), work schedule, anxiety/depression (Hospital Anxiety and Depression Scale), insomnia (Bergen Insomnia Scale), sleepiness (Epworth Sleepiness Scale) and fatigue (Fatigue Questionnaire). They were also asked to report number of night shifts in the last 12 months (NNL). First, the parameters were compared between nurses i) never working nights, ii) currently working nights, and iii) previously working nights, using binary logistic regression analyses. Subsequently, a cumulative approach was used investigating associations between NNL with the continuous scores on the same dependent variables in hierarchical multiple regression analyses.

**Results:**

Nurses with current night work were more often categorized with insomnia (OR = 1.48, 95% CI = 1.10–1.99) and chronic fatigue (OR = 1.78, 95% CI = 1.02–3.11) than nurses with no night work experience. Previous night work experience was also associated with insomnia (OR = 1.45, 95% CI = 1.04–2.02). NNL was not associated with any parameters in the regression analyses.

**Conclusion:**

Nurses with current or previous night work reported more insomnia than nurses without any night work experience, and current night work was also associated with chronic fatigue. Anxiety, depression and sleepiness were not associated with night work, and no cumulative effect of night shifts during the last 12 months was found on any parameters.

## Introduction

Shift work has been reported to be associated with various mental complaints, including anxiety, depression, insomnia and fatigue [1], [2], [3], [4], [5], [6], [7], [8]. Scott and colleagues found night work to be a risk factor for major depressive disorder, especially for women [5]. In that study, employees with more than 5 years of night work experience had more than a 6-fold increased risk of having a depressive disorder, compared with employees with 5 years or less of night work experience. A large prospective study from the UK found shift work to be associated with subsequent depression in both men and women [2]. Women's mental health was most impaired by unstable shift schedules, while night work had the strongest negative impact on men. A prospective study among female student nurses found night work to be negatively associated with psychological well-being 15 months after undertaking work [9]. On the other hand, there are also some studies that have not found any association between shift work and mental health [10], [11]. Additionally, any type of shift work can cause sleep loss, but it is especially pronounced in shift schedules including night shifts, since employees following night shifts go to bed when their diurnal rhythm promotes wakefulness [12], [13]. Indeed, disrupted sleep is reported to be the most challenging symptom among shift workers [14], and some studies report positive associations between former night work and chronic insomnia [15], [16]. Importantly, the impact of night work on physical and mental health has been reported to differ across study contexts [17]. This might explain some of the divergent findings between different studies.

To this date, most studies investigating health impacts of night work have used a categorical approach, i.e. comparing symptoms in night working employees with day working employees. Few studies have regarded night work as a continuous variable, which allows for the investigation of a possible dose-response or cumulative effects of night work on mental disorders and complaints. Against this backdrop we used data from a large sample of nurses in Norway, with the following aims:

1. To compare levels of anxiety, depression, insomnia, sleepiness and fatigue between nurses belonging to the following three groups: i) no night work experience ii) current night work, iii) previous night work.
2. To investigate whether there is a cumulative effect of number of night shifts during the last 12 months on the aforementioned parameters.

## Methods

### Ethics Statement

Ethical approval was given by the Regional Committee for Medical and Health Research West (REK) in Norway.

### Procedure

This study is a part of “The SUrvey of Shift work, Sleep and Health” (SUSSH), which investigates various aspects of shift work and other working conditions among Norwegian nurses. Invitations were initially sent to 6000 registered members of the Norwegian Nurses Organization (NNO) during the period December 2008 to March 2009, divided into five strata according to time since graduation [18]. As 600 letters were returned due to wrong addresses, a total of 5400 nurses actually received invitations to participate. A total of 2059 nurses participated, yielding a response rate of 38.1%. Among these, 2035 nurses answered questions about current or previous night work experience.

### Questionnaires

The questionnaire included items covering demographic factors (gender, age, marital status, children living at home) and working conditions (including years of nursing experience and estimated number of night shifts in the last 12 months – NNL). The questionnaire also included a question on current shift schedule (day-/evening shifts, only day shifts, only evening shifts, only night shifts, three shift schedule, other shift schedule including night shifts) and a question about having current or had previous night work (yes, no). Sociodemographic variables were chosen based on their possible impact on the perceived burden of night work or based on their possible association with mental complaints.

### Hospital Anxiety and Depression Scale

Symptoms of anxiety and depression were assessed by the Hospital Anxiety and Depression Scale (HADS), which is widely used in epidemiological studies [19], [20]. The questionnaire contains seven items reflecting nonvegetative anxiety symptoms (HADS-A) and seven items pertaining to nonvegetative depressive symptoms (HADS-D) experienced the last week. Each item is scored on a Likert scale (0–3), yielding a maximum score of 21 on both scales. A cut-off point of ≥8 was used to define possible cases of anxiety and depression based on the respective scales, as this yields a relatively good balance between specificity and sensitivity (both being approximately 0.8) [20]. Cronbachs alfa was .82 for both the anxiety and depression subscales, respectively.

### Bergen Insomnia Scale

The Bergen Insomnia Scale (BIS) was used to measure insomnia symptoms [21]. It is based on the Diagnostic and Statistics Manual for Mental Disorders, 4^th^ edition (DSM-IV) insomnia criteria [22], and asks about insomnia symptoms experienced during the last month. The scale contains six items, each with possible scores from 0 to 7 (corresponding to the number of days per week the actual symptom was experienced), yielding a possible range of the composite score from 0 to 42. The first four items refer to sleep onset, maintenance, early morning awakening and non-restorative sleep, while the two latter items reflect consequences of bad sleep (daytime impairment and dissatisfaction with sleep). Insomnia caseness is defined as scoring ≥3 on at least one of the first four items as well as ≥3 on at least one of the last two items [21]. Cronbachs alpha was .83 for the BIS questionnaire.

### Epworth Sleepiness Scale

Sleepiness was assessed using the Epworth Sleepiness Scale (ESS); the most commonly used self-report measure of sleepiness. The respondents are asked about their perceived tendency to fall asleep or to doze off in eight different situations on a scale ranging from 0 (would never doze off) to 3 (a high risk of dozing) [23]. A sum score exceeding 10 is considered as excessive sleepiness. Cronbachs alpha was .74 for the questionnaire.

### Fatigue Questionnaire

Fatigue was assessed by the Fatigue Questionnaire (FQ), an instrument commonly used to detect cases of chronic fatigue in general population studies [24]. The questionnaire has 13 items. The first 11 items have four response alternatives scored on a Likert scale (0–3), ranging from none to severe symptoms. The first seven items reflect physical fatigue, while the latter four assess mental fatigue (total range 0–33). The last two items measure persistence and duration of symptoms. In the present study an error was made regarding question 11 and answers were therefore handled by the ordinary rules for substitution. To define chronic fatigue, each of the first 11 items was dichotomized. The first two response alternatives were scored as 0, whereas the two latter response alternatives were scored as 1. Chronic fatigue cases were defined as scoring 1 on at least 4 of the 11 items and scoring ”six months or more” on item 12 (which concerns the duration of symptoms) [24]. The Cronbach's alpha was .89 for the physical fatigue scale and .84 for the mental fatigue scale.

### Statistics

Nurses were divided in three groups defined by their experience with night work (no night work experience, current night work, previous night work). This was done using the questions on current shift schedule and any current/previous night work experience. Nurses answering “no” to the question regarding having current or had previous night work were defined as having no night work experience, while nurses reporting a current shift schedule including night shift were defined as current night workers. Finally, nurses answering “yes” to the question on current or previous night work and reporting a current shift schedule not involving night work were defined as previous night workers. Some nurses both answered “no” to the question on current or previous night work experience and simultaneously reported night shifts during the last year (NNL>0). They were categorized as current night workers.

In terms of analyses we initially explored differences in demographic variables (age, gender, years of work experience, marital status, children living at home) and caseness of anxiety, depression, insomnia, excessive sleepiness and chronic fatigue between the three groups defined on basis of their relation to night work. This was done by using binary logistic regression analyses, hence caseness of anxiety, depression, insomnia, excessive sleepiness and chronic fatigue, respectively comprised the dependent variables. Each of the three defined groups was dummy coded (to a value of 1 or 0) and used as independent variables. No night work experience was used as the reference category. Crude analyses were performed, and in addition analyses with adjustment for age, gender, and years of work experience, marital status and children living at home (adjusted analyses).

Hierarchical linear multiple regression analyses were subsequently performed using two steps [25]. Total scores on HADS-A, HADS-D, BIS, ESS and FQ were used as dependent variables. Since both HADS-A, HADS-D and BIS scores violated the assumptions of normality, these variables were transformed using square root transformation before they were entered into the model. The ESS and FQ total scores did not violate the assumptions of normality. In the first step, we entered demographic and background variables (age, years of experience as nurse, marital status, and children living at home), whereas number of night shifts in the last 12 months (NNL) was entered in the second step. The study sample was stratified by (1) gender (men vs. women) and (2) number of years with night work experience (<3 years vs. ≥3 years). A two-tailed significance level of .05 was used, and all data were analysed using Statistical Package of Social Sciences (SPSS) version 20.0.

## Results

### Demographic characteristics (Table 1)

In total, 229 nurses reported no night work experience, 1315 nurses reported current night work experience, and 491 nurses reported previous (but no current) night work experience.

Nurses with no night work experience were less often married and had less often children living at home than nurses reporting current night work. Nurses reporting previous night work were older than the two other groups, were more often married and had more often children living at home than nurses reporting current night work or no night work experience (Table 1).

**Table 1:** Demographic characteristics of nurses with different night work status (no night work experience, current night work, previous night work).

	No night work experience (n = 229)	Current night work (n = 1315)	Previous night work (n = 491)
Age	**32.4 (31.2–33.6)**	**32.4 (32.0–32.8)**	**35.3 (34.5–36.1)**
Gender (% female)	**91.2 (87.5–94.9)**	**90.8 (89.2–92.3)**	**90.2 (87.5–92.8)**
Years of experience	**3.5 (2.9–4.0)**	**5.1 (4.9–5.4)**	**6.0 (5.6–6.4)**
Marital status (% married/cohabiting)	**68.6 (62.5–74.6)**	**73.5 (71.1–75.9)**	**76.4 (72.7–80.2)**
Children living at home (% yes)	**42.9 (36.3–49.5)**	**48.7 (45.9–51.4)**	**59.4 (54.9–63.9)**
HADS–Anxiety score	**4.5 (4.1–5.0)**	**4.7 (4.5–4.9)**	**4.7 (4.4–5.0)**
Anxiety Cases (%)	**19.1 (13.9–24.3)**	**19.7 (17.5–21.9)**	**21.0 (17.3–24.7)**
HADS–Depression score	**2.4 (2.1–2.7)**	**2.8 (2.7–3.0)**	**2.9 (2.6–3.2)**
Depression Cases (%)	**6.2 (3.0–9.4)**	**8.8 (7.2–10.3)**	**10.5 (7.8–13.3)**
Bergen Insomnia Scale	**12.5 (11.4–13.6)**	**13.6 (13.2–14.1)**	**13.8 (13.0–14.6)**
Insomnia Cases (%)	**46.9 (40.4–53.5)**	**55.0 (52.3–57.7)**	**54.1 (49.7–58.5)**
Epworth Sleepiness Scale	**8.1 (7.6–8.6)**	**8.6 (8.4–8.8)**	**8.2 (7.8–8.5)**
Sleepiness Cases (%)	**25.8 (19.9–31.7)**	**29.6 (27.1–32.2)**	**25.6 (21.7–29.6)**
FQ total score	**13.1 (12.6–13.6)**	**13.7 (13.4–13.9)**	**13.7 (13.3–14.1)**
Fatigue Cases (%)	**8.0 (4.4–11.5)**	**12.8 (10.9–14.6)**	**13.0 (10.0–16.0)**

The results are reported as mean values with 95% confidence intervals in parentheses.

HADS = Hospital Anxiety and Depression Scale.

FQ = Fatigue Questionnaire.

In the total sample, mean age was 33.1 years (range 21–63 years, SD = 8.2), 90.6% of the respondents were women, and 55.8% were full-time employees (defined as having at least a 90% full time equivalent position). Mean years of experience as nurse were 5.2 years (range 0–37 years SD = 4.3), 67.3% reported their current shift schedule to include night work, and mean number of night shifts the last year was 25.6 nights (95% CI = 24.4–26.9). Overall, 73.7% of the nurses were married/cohabiting, and 50.5% had children living at home. A total of 76.0% worked in somatic hospital departments, 13.8% worked in psychiatric hospital departments, while the remaining nurses worked either in nursing homes, home care services, public health services or other services. Caseness criteria for anxiety were fulfilled by 19.9% (95% CI = 18.1–21.6), while caseness criteria for depression were fulfilled by 8.8% (95% CI = 7.6–10.1). Caseness criteria of insomnia were fulfilled by 53.9% (95% CI = 51.7–56.0), while excessive sleepiness and chronic fatigue caseness were found in 28.3% (95% CI = 26.4–30.3) and in 12.3% (95% CI = 10.8–13.7) of the sample, respectively.

### Comparison of nurses with current, previous or no night work experience (Table 2)

In the crude analyses, insomnia and chronic fatigue caseness were positively associated with current night work compared with no night work experience. In the adjusted analyses, both current and previous night work were positively associated with insomnia caseness, while current night work was positively associated with chronic fatigue caseness compared with no night work experience.

**Table 2:** Logistic regression analyses using caseness of anxiety, depression, insomnia, excessive sleepiness and chronic fatigue as dependent variables, and night work status (no night work experience, current night work, previous night work) as independent variables.

	Crude					Adjusted*				
	Anxiety	Depression	Insomnia	Excessive sleepiness	Chronic fatigue	Anxiety	Depression	Insomnia	Excessive sleepiness	Chronic fatigue
No night work experience	**1.00**	**1.00**	**1.00**	**1.00**	**1.00**	**1.00**	**1.00**	**1.00**	**1.00**	**1.00**
Current night work	**1.04** **(.73–1.49)**	**1.46** **(.82–2.59)**	**1.38** **(1.04–1.83)**	**1.21** **(.87–1.68)**	**1.69 (1.02–2.81)**	**1.18** **(.81–1.74)**	**1.35** **(.75–2.42)**	**1.48** **(1.10–1.99)**	**1.27** **(.90–1.80)**	**1.78** **(1.02–3.11)**
Previous night work	**1.13** **(.76–1.68)**	**1.78** **(.96–3.29)**	**1.33** **(.97–1.83)**	**.99** **(.69–1.43)**	**1.73 (1.00–3.00)**	**1.27** **(.82–1.94)**	**1.53** **(.81–2.87)**	**1.45** **(1.04–2.02)**	**1.12** **(.76–1.65)**	**1.69** **(.93–3.09)**

The independent variables were inserted simultaneously, and no night work experience was used as the reference category. Results are reported as odds-ratios, and 95% confidence intervals are shown in parentheses. Underlined values are statistically significant.

* Adjusted analyses were adjusted for age, gender, and years of work experience, marital status and children living at home.

### Anxiety and depression (Table 3)

In the hierarchical multiple regression analyses among women, there was a negative association between years of work experience as a nurse and the anxiety score, while age was positively associated with the depression score. Having children was positively associated with the depression score in women. Age was positively associated with the depression score only in nurses with less than 3 years of night work experience.

**Table 3:** Hierarchical multiple regression analyses using two steps.

	Anxiety score		Depression score		Insomnia score		Sleepiness score		Fatigue score	
**Part 1A – Men (n = 192)**										
	*β*	*Δ*R2	*β*	*Δ*R2	*β*	*Δ*R2	*β*	*Δ*R2	*β*	*Δ*R2
**Step 1**		.027		.010		.009		.005		.012
**Age**	−.126		.084		−.063		−.054		.070	
**Years of experience**	−.012		.001		.019		−.029		−.017	
**Married/Cohabiting**	−.050		.056		−.035		−.015		.007	
**Children**	−.051		−.010		−.047		.005		−.098	
**Step 2**		.018		.011		.002		.003		.007
**Age**	−.143		.071		−.068		−.061		.060	
**Years of experience**	−.014		.000		.019		−.030		−.018	
**Married/Cohabiting**	−.051		.056		−.036		−.016		.006	
**Children**	−.034		.003		−.041		.012		−.088	
**Number of nights last year**	.137		.107		.049		.055		.083	
**Part 1B – Women (n = 1857)**										
	*β*	*Δ*R2	*β*	*Δ*R2	*β*	*Δ*R2	*β*	*Δ*R2	*β*	*Δ*R2
**Step 1**		.007*		.009*		.002		.011*		.004
**Age**	.015		.071*		.033		−.058*		.003	
**Years of experience**	−.062*		−.023		−.013		−.023		−.016	
**Married/Cohabiting**	−.014		−.046		−.015		−.020		−.049	
**Children**	−.050		.061*		.023		−.046		.057*	
**Step 2**		.175		.001		.000		.000		.000
**Age**	.011		.074*		.033		−.056		.003	
**Years of experience**	−.058*		−.027		−.013		−.024		−.016	
**Married/Cohabiting**	−.015		−.045		−.015		−.020		−.049	
**Children**	−.052		.062*		.023		−.046		.057*	
**Number of nights last year**	−.033		.028		.002		.009		.002	
**Part 2A – <3 years work experience (n = 654)**										
	*β*	*Δ*R2	*β*	*Δ*R2	*β*	*Δ*R2	*β*	*Δ*R2	*β*	*Δ*R2
**Step 1**		.008		.015		.005		.023*		.006
**Age**	.006		.117*		.087		.013		.033	
**Years of experience**	−.051		−.040		−.014		−.075		.002	
**Married/Cohabiting**	−.013		−.055		.016		.021		−.021	
**Children**	−.058		.034		−.053		−.127*		.058	
**Step 2**		.002		.001		.000		.000		.003
**Age**	.000		.120*		.089		.014		.025	
**Years of experience**	−.055		−.037		−.012		−.074		−.004	
**Married/Cohabiting**	−.015		−.054		.017		.022		−.023	
**Children**	−.060		.035		−.052		−.127*		.056	
**Number of nights last year**	−.045		.027		.018		.011		−.057	
**Part 2B – ≥3 years night work experience (n = 1096)**										
	*β*	*Δ*R2	*β*	*Δ*R2	*β*	*Δ*R2	*β*	*Δ*R2	*β*	*Δ*R2
**Step 1**		.007		.007		.001		.010*		.006
**Age**	.010		.063		−.012		−.084*		−.014	
**Years of experience**	−.030		−.001		.012		.006		−.033	
**Married/Cohabiting**	−.020		−.044		−.024		−.037		−.071*	
**Children**	−.065		.050		.028		−.026		.016	
**Step 2**		.000		.001		.001		.000		.000
**Age**	.009		.064		−.013		−.084*		−.014	
**Years of experience**	−.029		−.002		.013		.007		−.034	
**Married/Cohabiting**	−.021		−.043		−.025		−.038		−.071*	
**Children**	−.067		.053		.024		−.028		.018	
**Number of nights last year**	−.016		.027		−.028		−.017		.011	

Men (n = 192) The first step contained the adjusting variables age, years of experience as nurse, married/cohabiting (0 = no, 1 = yes) and children living at home (0 = no, 1 = yes), while the second step included number of night shifts in the last 12 months. Total scores on anxiety (HADS-A), depression (HADS-D), insomnia (BIS), sleepiness (ESS) and fatigue (FQ) were used as dependent variables. Anxiety, depression and insomnia variables were transformed using square root transformation before they were entered into the model. Results are reported as standardized beta-coefficients (*β*) and the R square changes (*Δ*R2) (n = 2035). Analyses are stratified for (1) men and women and (2) night work experience less than three years vs. at least three years.

HADS = Hospital Anxiety and Depression Scale (A = anxiety subscale, D = depression subscale).

BIS = Bergen Insomnia Scale.

ESS = Epworth Sleepiness Scale.

FQ = Fatigue Questionnaire.

* p<.05.

No significant associations were found in the hierarchical multiple regression analyses for men or any nurses with at least 3 years of night work experience, and number of night shifts last 12 months (NNL) was not associated with any of the parameters.

### Insomnia, sleepiness and fatigue (Table 3)

In the hierarchical multiple regression analyses among women, age was negatively associated with the sleepiness score in the first step of the analysis, but this association was no longer significant in the second step. Among nurses with at least 3 years of night work experience however, age was negatively associated with sleepiness in both steps of the analysis. Being married/cohabiting was negatively associated with fatigue in women, while having children was negatively associated with sleepiness in subjects with less than 3 years of night work experience.

No significant associations were found in the hierarchical multiple regression analyses for men, and number of night shifts last 12 months (NNL) was not associated with any of the parameters.

## Discussion

This study shows that nurses with current or previous night work experienced more caseness of insomnia than nurses with no night work experience. Current night work was also associated with chronic fatigue caseness. There was no evidence for a cumulative relationship between the number of night shifts the last 12 months and neither insomnia, sleepiness nor fatigue. Furthermore, there were no associations between night work, anxiety and depression in neither the categorical nor the cumulative approaches.

Hence, our results suggest that anxiety and depression are not associated with the presence or extent of night work. Our finding is in accordance with some previous studies [10], [11] but in contrast with others [2], [5]. As mentioned in the methods section, however, there was a noticeable number of non-responders, which should be taken into account and prompts for a careful interpretation of the results. A previous Norwegian health survey has shown serious psychiatric morbidity to be more prevalent among non-attenders than among attenders [26]. This may explain why no relationship between anxiety and depression and night work was found in the present study. The different results across studies could also rely on differences in the populations studied, differences in response rate and differences in the questionnaires used. Our study deviates from previous studies, since we included both a categorical and a cumulative approach, strengthening the reliability of the results.

Still, our data could be affected by selection bias. There has been a considerable concern that much research on night work is influenced by a “healthy worker” effect. This reflects that employees struggling with night work are likely to stop working nights early in their career. Consequently, long-term night workers represent a relatively healthy part of the population. Such selection effects are widely known in research addressing the health effects of shift work [27].

Using the Bergen Insomnia Scale (BIS), we found significantly more insomnia caseness among nurses with current or previous night work compared with nurses with no night work experience. Associations between current night work and insomnia could reflect the fact that employees with night work often go to bed when the diurnal rhythm promotes wakefulness. This usually results in shortened sleep duration, which is experienced as undesirable by the employees. It may be somewhat problematic to administer an insomnia scale to night shift workers as it is currently not known whether insomnia for a night worker equals insomnia for other individuals [28]. Our results might also suggest that night work induced insomnia is not completely reversed when employees stop working nights. However, nurses with chronic sleep problems might be more interested than other nurses in reporting their symptoms in a study on health and sleep, and this might give a bias in our study. In addition, the high number of non-responders may have influenced the results, but we do not know the effect of this possible non-responder bias. There are few studies investigating the relationship between shift work and the development of chronic insomnia using longitudinal designs. A study on a group of day-working nurses reported most insomnia symptoms in nurses with a history of more than five night shifts per months for at least four years [15]. Additionally, a retrospective cohort twin study from Sweden found that previous night work was significantly associated with current disturbed sleep and poorer self-rated health [16]. In this respect it is interesting to note that among nurses with ≥3 years of night work experience, age was negatively associated with sleepiness. It is possible that employees acquire coping strategies over the years, minimizing adverse effects of night work on sleep. Another possible explanation is that these results are a result of the “healthy worker effect” mentioned earlier. However, the coefficient is rather small and the clinical significance is therefore unclear.

We found a negative association between being married/cohabiting and fatigue in nurses with at least three years of night work experience, and a negative association between having children and sleepiness in nurses with less than 3 years of night work experience. The former finding might be expected, while the latter is more difficult to account for. A possible, but speculative explanation is that nurses with children in their early career have a tendency to develop insomnia [29]. As many insomniacs are characterized by hyperarousal they typically report low scores on sleepiness [23], [30]. We also found that female nurses with children reported more fatigue and more depression than other female nurses, but this coefficient was again rather small.

Surprisingly, we did not find significantly elevated levels of sleepiness in nurses with current night work compared with the other groups. The mean Epworth Sleepiness Scale score in all the groups in the current study was higher than commonly reported mean scores from the general population, which usually vary between 4.5–7.0 [31], [32]. A previous telephone survey reported that the mean score of the ESS in the Norwegian population is 7.4 for men and 6.5 for women [33]. Hence, a higher than average level of sleepiness in all groups could mask a possible effect of current night work on sleepiness, and could as such represent a ceiling effect. In this context, it should be borne in mind that most nurses in the reference group were also shift workers, although they did not work nights. There was nevertheless no significant dose-response relationship between number of night shifts during the last year and sleepiness.

### Strengths and limitations

This is so far one of the largest studies investigating the associations between night work and symptoms of mental disorders. In addition, the cumulative approach represents a new and useful way to strengthen the validity of our conclusions.

The cross-sectional design of this study represents however an important limitation. There are many practical and familial reasons to quit night work, not only the fact that night work might be experienced as distressing. We are prohibited from exploring causal relationships in the present study, and a prospective study is necessary in order to truly investigate whether night work increases the risk of mental disorders or not.

All participants of the present study were nurses, restricting the range of both socioeconomic status and work content. Thus, generalization to the general shift work population might be limited, although currently there is no good reason to believe that nurseś psychobiological response to night work is very different from other night working employees. Another limitation was the high number of females in the population, making our results not representative for male dominated working populations. The low number of male nurses might also account for the lack of significant relationships in the analyses in the male subgroup. The mean age of our study population was relatively low. This could possibly affect our results, since more experienced nurses might have developed more resilient coping strategies to counteract stressful working conditions including night work. A strength, however, is that a low mean age minimizes the possible detrimental effects of menopause on sleep.

There were a high number of non-responders in our material, which is a considerable limitation in many comorbidity studies. It could be argued that nurses experiencing night work as a problem were more willing to participate than nurses not experiencing difficulties with night work.

## Conclusions

Among nurses, current or previous night work experience was associated with more caseness of insomnia than no night work experience. Current night work was associated with chronic fatigue caseness. There were no significant relationships between night work and caseness of anxiety, depression or sleepiness. No cumulative effect of night shifts during the last 12 months was found on any parameters.
